# Encapsulated *Brucella ovis* Lacking a Putative ATP-Binding Cassette Transporter (Δ*abcBA*) Protects against Wild Type *Brucella ovis* in Rams

**DOI:** 10.1371/journal.pone.0136865

**Published:** 2015-08-28

**Authors:** Ana Patrícia C. Silva, Auricélio A. Macêdo, Luciana F. Costa, Cláudia E. Rocha, Luize N. N. Garcia, Jade R. D. Farias, Priscilla P. R. Gomes, Gustavo C. Teixeira, Kessler W. J. Fonseca, Andréa R. F. Maia, Gabriela G. Neves, Everton L. Romão, Teane M. A. Silva, Juliana P. S. Mol, Renata M. Oliveira, Márcio S. S. Araújo, Ernane F. Nascimento, Olindo A. Martins-Filho, Humberto M. Brandão, Tatiane A. Paixão, Renato L. Santos

**Affiliations:** 1 Departamento de Clínica e Cirurgia Veterinárias, Escola de Veterinária, Universidade Federal de Minas Gerais, 31270–901 Belo Horizonte, MG, Brazil; 2 Departamento de Patologia Geral, Instituto de Ciências Biológicas, Universidade Federal de Minas Gerais, 31270–901 Belo Horizonte, MG, Brazil; 3 Departamento de Patologia, Universidade Estadual do Maranhão, 65057–630 São Luís, MA, Brazil; 4 Centro de Pesquisas René Rachou, Fundação Oswaldo Cruz, 30190–002 Belo Horizonte, MG, Brazil; 5 Embrapa Gado de Leite, 36038–330 Juiz de Fora, MG, Brazil; Institut National de la Recherche Agronomique, FRANCE

## Abstract

This study aimed to evaluate protection induced by the vaccine candidate *B*. *ovis ΔabcBA* against experimental challenge with wild type *B*. *ovis* in rams. Rams were subcutaneously immunized with *B*. *ovis* Δ*abcBA* encapsulated with sterile alginate or with the non encapsulated vaccine strain. Serum, urine, and semen samples were collected during two months after immunization. The rams were then challenged with wild type *B*. *ovis* (ATCC25840), and the results were compared to non immunized and experimentally challenged rams. Immunization, particularly with encapsulated *B*. *ovis* Δ*abcBA*, prevented infection, secretion of wild type *B*. *ovis* in the semen and urine, shedding of neutrophils in the semen, and the development of clinical changes, gross and microscopic lesions induced by the wild type *B*. *ovis* reference strain. Collectively, our data indicates that the *B*. *ovis* Δ*abcBA* strain is an exceptionally good vaccine strain for preventing brucellosis caused by *B*. *ovis* infection in rams.

## Introduction

Brucellosis is an infectious disease with worldwide distribution. It is caused by *Brucella* spp., which infects domestic and wild animals, and humans [1], causing significant economic losses [2]. *Brucella* spp. are Gram negative, uncapsulated and immobile bacilli that belong to the α2-Protobacteriacea family [3]. *Brucella ovis* does not cause human disease, but it induces chronic infection in sheep [4].

The most common clinical manifestations of *B*. *ovis* infections are epididymitis in rams and occasional abortion in ewes [5–7]. Therefore, due to losses caused by *B*. *ovis*-induced infertility, research efforts have been focusing on the development of novel vaccines for controlling *B*. *ovis* infection [8–11].

The most commonly used vaccine against brucellosis in small ruminants is the *B*. *melitensis* Rev1 strain. This live attenuated vaccine provides good levels of protection against *B*. *melitensis* in sheep and goats [12–14], and induces cross protection against *B*. *ovis* in sheep [15]. However, the Rev1 strain has pathogenic potential, being capable to infect and cause disease in humans and to cause abortion in ewes. Furthermore, Rev1 is resistant to streptomycin [16,17], and it interferes with routinely used serological assays [18]. Importantly, Rev1 cannot be used in *B*. *melitensis*-free areas such as in Brazil [19].

Research conducted over the past 100 years has demonstrated that the best brucellosis vaccination strategy is the use of live attenuated vaccine strains [20–22]. A mutant *B*. *ovis* strain lacking a predicted ABC transporter (*B*. *ovis* Δ*abcBA*) is attenuated in mice, indicating that this live attenuated strain may be a vaccine candidate against *B*. *ovis* infection in rams [23].

*B*. *ovis* Δ*abcBA* strain induces humoral and cellular responses that are similar to those triggered by wild type infection, whereas in contrast to the wild type strain, *B*. *ovis* Δ*abcBA* is not shed in the semen and urine of experimentally infected rams [24]. Recent data from our laboratory demonstrated that alginate encapsulated *B*. *ovis* Δ*abcBA* induces protection against experimental challenge in mice, decreasing bacterial loads in the spleen and preventing lesions [25]. These recent results encouraged us to evaluate the *B*. *ovis* Δ*abcBA* strain as a live attenuated vaccine strain in rams.

Therefore, the aim of this study was to evaluate the protective and immunogenic potential of the *B*. *ovis* Δ*abcBA* strain, either encapsulated with alginate or non encapsulated, against experimental challenge with wild type *B*. *ovis* in rams.

## Material and Methods

### Bacterial strains and culture conditions

*B*. *ovis* ATCC 25840 (wild type strain), *B*. *ovis* Δ*abcBA*, which has been previously described [23], and mCherry-expressing *B*. *ovis* Δ*abcBA* [26] were used in this study. Inocula were grown on Tryptose Soy Agar (TSA) with 1% hemoglobin (Becton Dickinson, Brazil), for 3 days at 37°C, in a humidified 5% CO_2_ atmosphere, and then suspended in sterile PBS (phosphate buffered saline). Inocula concentration was estimated by spectrophotometry at an optical density of 600 nm (OD_600_). For *B*. *ovis* Δ*abcBA* culture, 100 mg/mL kanamicin (Bio-Rad, Hercules, USA) was added to TSA medium with 1% hemoglobin.

### *Brucella ovis* ΔabcBA encapsulation

Encapsulation of *B*. *ovis* Δ*abcBA* strain was performed as previously described [25]. Briefly, a suspension containing 1 x 10^11^ CFU of *B*. *ovis* Δ*abcBA* was added to sodium alginate (Sigma—Aldrich, Brazil), and then this mixture was placed in a syringe and dripped with a 0.23 x 4 mm needle into a 100 mM CaCl_2_ solution. After dripping, capsules were formed and then homogenized. Capsules were washed in MOPS buffer solution (Sigma—Aldrich, Brazil), followed by addition of poly-L-lysine solution (Sigma—Aldrich, Brazil) for 15 min under agitation, and then washed in MOPS buffer. Particles were added to alginate solution during 5 min, and then suspended in MOPS buffer. Particles were inoculated subcutaneously in the final dose of 10^9^ CFU per ram. Particle sizes were assessed by light microscopy and scanning electron microscopy. Effectiveness of bacterial encapsulation and density was assessed by encapsulating mCherry-expressing *B*. *ovis* **Δ** *abcBA* followed by fluorescence microscopy (Leica DM 4000 B) as previously described [25]. For tridimensional evaluation by scanning electron microscopy (SEM), alginate capsules were attached to glass cover slips pretreated with 0.1% of poly-L-lysinehydrobromide solution (Sigma—Aldrich, USA). Samples were fixed in 2.5% glutaraldehyde solution in 0.1 M phosphate buffer pH 7.2 for 2 h at 4°C, followed by secondary fixation in sequential solution of osmium, tannic acid, and osmium, dehydration in ethanol, and critical point drying in a CO_2_ dryer. Then, cover slips were mounted on SEM stubs, sputter coated with gold, and viewed with Zeiss DSM950 SEM at accelerating voltage of 13 kV.

### Rams

Thirty crossbreed 1-year-old *B*. *ovis*-free rams were used in this study. Negativity to *B*. *ovis* infection was based on serology and PCR as described below. Rams were fed hay and concentrate with 18% crude protein twice a day. They received water and ovine mineral mixture *ad libitum*. Rams were conditioned for semen sampling using a *B*. *ovis*-free ewe during three weeks, and then they were randomly divided into three groups (10 rams per group) in completely separated and independent premises, with different handlers and with no direct or indirect contact between these groups.

Ten rams were inoculated subcutaneously with 2 mL PBS, other 10 rams were immunized subcutaneously with 2 mL of a suspension containing 1 x 10^9^ CFU of *B*. *ovis* Δ*abcBA* strain, and the other 10 were immunized with *B*. *ovis* Δ*abcBA* encapsulated within sterile alginate capsules through the same route, volume and concentration used in the other vaccinated group.

Two months after vaccination, rams were challenged as previously described [27], with 2 mL of a suspension containing 1.2 x 10^9^ CFU/mL of ATCC25840 *B*. *ovis* strain intraprepucially, plus 50 μL in each conjunctival sac of a solution containing 1.2 x 10^10^ CFU/mL of the same strain (totaling 3.6 x 10^9^ CFU).

Blood, semen, and urine samples were collected every two weeks for 2 months immediately before and two months after challenge. These samples were used for AGID, lymphocyte proliferation assay, leukocyte immunophenotyping, semen smear, bacterial culture, and PCR.

Two months after challenge, rams were sedated with xylazine (Copazine, Schering-Plough Coopers, Brazil), deeply anesthetized with sodium thiopental (Cristalia, Brazil), and then euthanatized by electrocution, which was immediately followed by necropsy. Fragments of the tail, head, and body of the epididymis, testes, vesicular gland, bulbourethral gland, glans, penis, prepuce, iliac lymph nodes, spleen, and liver were collected and processed for bacterial culture and DNA extraction followed by PCR. This experiment was approved by the Institutional Ethics Committee for Animal Experimentation of the Universidade Federal de Minas Gerais (CETEA/UFMG, protocol number 204/2012). During the course of the experiment, rams were evaluated by a veterinarian twice a day. Since the rams did not developed spontaneous signs of pain or depression, no analgesic therapy was administered.

### Serology (agar gel immune diffusion—AGID)

A commercial AGID kit (TECPAR, Brazil) has been used according to the manufacturer’s instructions. Briefly, 4.6 mL of a 1% agarose solution (Invitrogen, Brazil) in 0.1 M borate buffer pH 8.0 with 1 M NaCl were laid onto a defatted glass slide. Seven holes forming a hexagonal shape (one central and six peripheral) were made. Serum samples were distributed in the peripheral holes alternately with the positive control serum, and the antigen was placed in the central hole. The slides were placed in a humidified chamber at room temperature and the reading was performed after 72 h of incubation.

### Bacterial culture

For bacterial isolation, 100 μL of urine or semen or tissues samples were aseptically plated on Thayer Martin agar with 1% hemoglobin. Tissue samples were transferred to 15 mL tubes containing 2 mL sterile PBS, then macerated with a tissue homogenizer (CB, Biotech, Brazil). To avoid contamination between samples, the homogenizer was washed twice with sterile water, followed by absolute ethanol, and then sterile water. For *B*. *ovis* Δ*abcBA* isolation from semen and urine of encapsulated and non-encapsulated *B*. *ovis* Δ*abcBA* immunized rams, 100 mg/mL kanamicin was added to Thayer Martin agar with 1% hemoglobin. Plates were incubated at 37°C, in humidified 5% CO_2_ incubator for 7 days.

### PCR

DNA extraction was performed using 500 μL of semen, 1 mL of urine, or approximately 500 μL of macerated thawed tissue samples, as previously described [28]. DNA was stored at -20°C until PCR analysis. Extracted DNA (100–500 ng) was added to 23 μL of a commercial PCR mix containing 22 mM Tris-HCl (pH 8.4), 55 mM KCl, 1.65 mM MgCl_2_, 220 μM of each dNTP (Supermix, Invitrogen, Brazil), 0.5 μL of each primer (GCCTACGCTGAAACTTGCTTTTG and ATCCCCCCATCACCATAACCGAAG) for a final concentration 25 μM [27] and additional 0.25 μL (27 U/μL) of Taq DNA polymerase (Invitrogen, Brazil). Amplification parameters were: 95°C for 5 min, followed by 30 cycles of 95°C for 1 min, 57°C for 1 min, and 72°C for 1 min, with a final step of extension at 72°C for 5 min. The expected PCR product had 228 base pairs. To differentiate *B*. *ovis* Δ*abcBA* strain from the wild type *B*. *ovis*, the following primes were used: GGCCCGGTTTTCTGTCTCAA and TCATCACGGTACTTGGGCTC, under the same conditions described above.

### Lymphocyte proliferation assay

Blood samples were collected at three time-points (immediately before immunization, 8 weeks after immunization, and 8 weeks after challenge). Lymphocyte proliferation was performed as previously described [24]. Briefly, blood was mixed to RPMI culture medium 1640 (Invitrogen, Brazil) at a ratio of 1:1 layered slowly onto a Histopaque 1077 (Sigma—Aldrich, Brazil) and centrifuged. After centrifugation, mononuclear cells were collected from the interface Histopaque/plasma and transferred to a 50 mL tube containing 40 mL of RPMI. Cells were centrifuged and subsequently, cell suspension was adjusted to 1 x 10^7^ cells/mL in RPMI. Cell suspension received 10 mM of the immunoproliferation marker CFSE (Carboxyfluorescein diacetate succinimidyl ester) and it was placed in flat-bottom 96-well plates (Corning, USA). Positive control wells received 25 μL of the mitogen PHA (Phythohemagglutinin), additional wells received 25 μL of a *B*. *ovis* antigen (5 μg/mL), and negative control wells received only supplemented RPMI 1640 medium. Plates were incubated at 37°C in a humidified incubator with 5% CO_2_ for five days. After this period, cells were transferred to polystyrene tubes and 30,000 events were counted by flow cytometer (FACSCalibur, Becton Dickinson, USA).

### Leukocyte immunophenotyping profile

At 1 and 4 weeks post immunization and 1 and 4 weeks post challenge, peripheral blood was sampled for immunophenotyping as previously described [24]. This assay was performed using specific anti-bovine monoclonal cell receptor antibodies known to cross-react with corresponded ovine antigens: anti-CD4 (Clone 44.38 –FITC—MCA2213F, AbD Serotec, USA), anti-CD8 (Clone CC63 –FITC—MCA 837F, AbD Serotec, USA), anti-CD21 (Clone CC21 –FITC—MCA 1424F, AbD Serotec, USA) and anti-γ/Δ (Clone CC15 –FITC—MCA838F, AbD Serotec, USA).

### Histopathology

Fragments of the tail, head, and body of the epididymis, testes, vesicular glands, bulbourethral glands, glans, penis, prepuce, iliac lymph nodes, spleen, and liver were fixed by immersion in 10% buffered formalin for 24 h, followed by dehydration in increasing concentrations of ethanol, xylene diaphanization, and paraffin embedding. Five μm-thick sections were stained with hematoxylin and eosin (HE) for histophatological evaluation.

### Immunohistochemistry

To verify intralesional localization of *B*. *ovis*, immunohistochemistry was performed as previously described [23]. Briefly, tissue sections were hydrated and incubated with 10% hydrogen peroxide in PBS for 30 min. After wash with PBS, slides were transferred to a humid chamber at room temperature, incubated with 25 mg/mL of skim milk for 45 min, and then incubated with primary antibody for 30 min. For immunolabeling, diluted serum (1:1,000) from a rabbit experimentally inoculated twice (at a 1-month interval) with 1 x 10^9^ CFUs of *B*. *ovis* (strain ATCC 25840) was used as primary antibody. Then tissue sections were washed with PBS, incubated with secondary antibody for 20 min, washed again with PBS, and incubated for 20 min with streptavidin-peroxidase from a commercial kit (LSAB kit; Dako Corporation, Carpinteria, USA). The reaction was revealed using 5% of 3-amino-9-etilcarbazol (AEC, Sigma—Aldrich, Brazil) and sections were counterstained with Mayer’s hematoxylin.

### Semen evaluation

To evaluate inflammatory cells in the semen, smears were stained with Quick Panoptic and examined under light microscopy. All semen samples were collected using an artificial vagina under aseptic conditions. Semen samples were immediately placed in a water bath at 37°C.

### Statistical analysis

Frequency of *B*. *ovis* detection by AGID, bacterial culture and PCR were compared by the Student-Newman-Keuls test (SNK). Lymphocyte proliferation data were compared by the SNK test, using GraphPad Prisma 5.0 software (GraphPad Prisma software, Inc 5.0, USA).

## Results

### Characterization of alginate capsules

**Fig 1:**
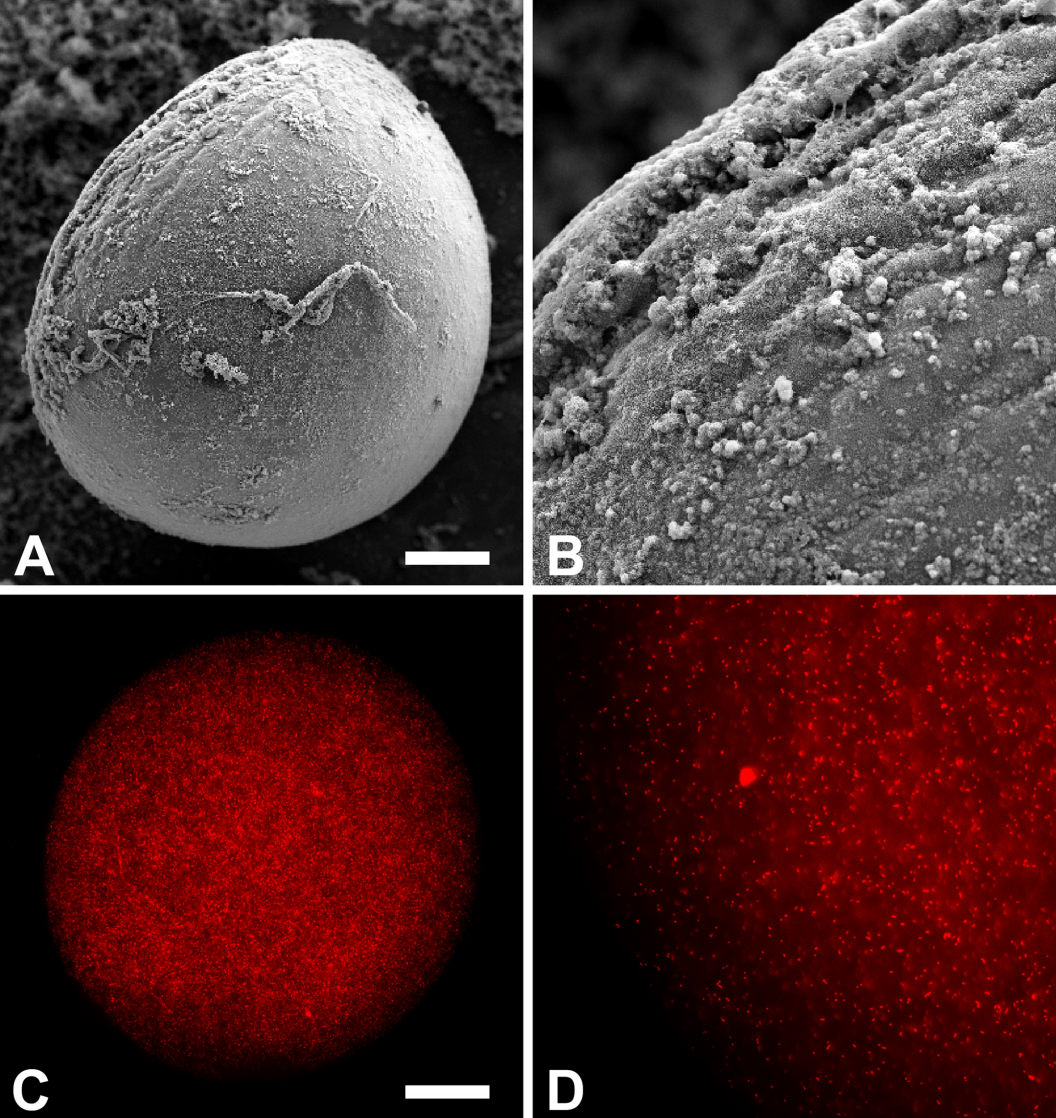
Scanning electron micrograph of alginate capsules, Bar = 100 μm (A); detail of the surface of the capsule (B); fluorescence microscopy of alginate capsules containing *mCherry*-expressing *Brucella ovis* Δ*abcBA*, Bar = 100 μm (C). Higher magnification demonstrating individualized *mCherry*-expressing *Brucella ovis* Δ*abcBA* (D).

Alginate capsules were evaluated under light microscopy (not shown) and scanning electron microscopy. Efficiency of *B*. *ovis* encapsulation was assessed by examining capsules containing *B*. *ovis* Δ*abcBA* expressing *mCherry* under fluorescence microscopy. Alginate capsules or capsules containing *B*. *ovis* were spherical or slightly oval and ranged from 300 to 800 μm in diameter, with similar shape (Fig 1A). Numerous red fluorescent *mCherry*-expressing *B*. *ovis* Δ*abcBA* were observed inside alginate capsules (Fig 1B).

### Humoral and cellular immune responses induced by encapsulated or non-encapsulated *Brucella ovis* ΔabcBA immunization

AGID was performed to evaluate the humoral response triggered by *B*. *ovis* Δ*abcBA*. Ninety percent (9/10) of rams immunized with encapsulated *B*. *ovis* Δ*abcBA* and 70% (7/10) of rams immunized with non-encapsulated mutant strain became serologically positive by AGID at two weeks after immunization. Both immunized groups remained seropositive until the eighth week post-immunization. As expected, non-immunized rams were not serologically positive for *B*. *ovis* before challenge.

**Fig 2:**
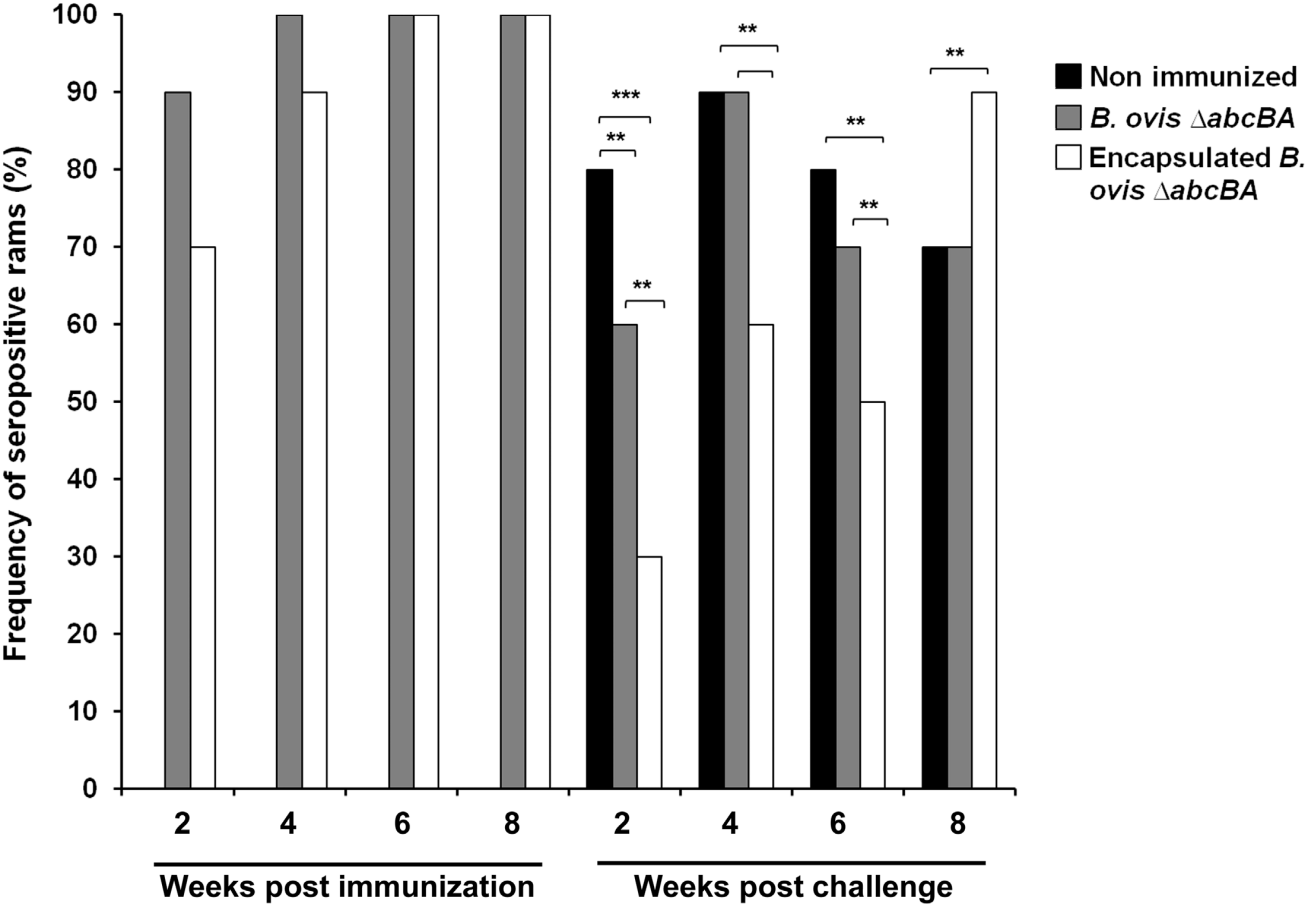
Frequency of seropositive rams (non immunized or immunized with encapsulated or non encapsulated *Brucella ovis* Δ*abcBA*). Seropositivity was determined by agar gel immune diffusion (AGID) before and after challenge. The number of weeks before and after challenge is indicated in the x axis. Statistical differences between groups (10 rams per group) are indicated by asterisks (**p<0.01; *** p<0.001).

During the first two weeks after challenge, there was a significant decrease in the frequency of seropositive rams that were immunized with encapsulated *B*. *ovis* Δ*abcBA* (3/10, p<0.001) or with non-encapsulated *B*. *ovis* Δ*abcBA* (6/10, p<0.05). During the following two weeks, the number of seropositive rams increased in both groups, to 90% in the group of rams immunized with encapsulated *B*. *ovis* Δ*abcBA* and to 60% in the group of rams immunized with non-encapsulated *B*. *ovis* Δ*abcBA*. At eight weeks after challenge, the percentages of seropositive rams were 90% and 70% for rams immunized with encapsulated or non-encapsulated *B*. *ovis* Δ*abcBA*, respectively (Fig 2).

**Fig 3:**
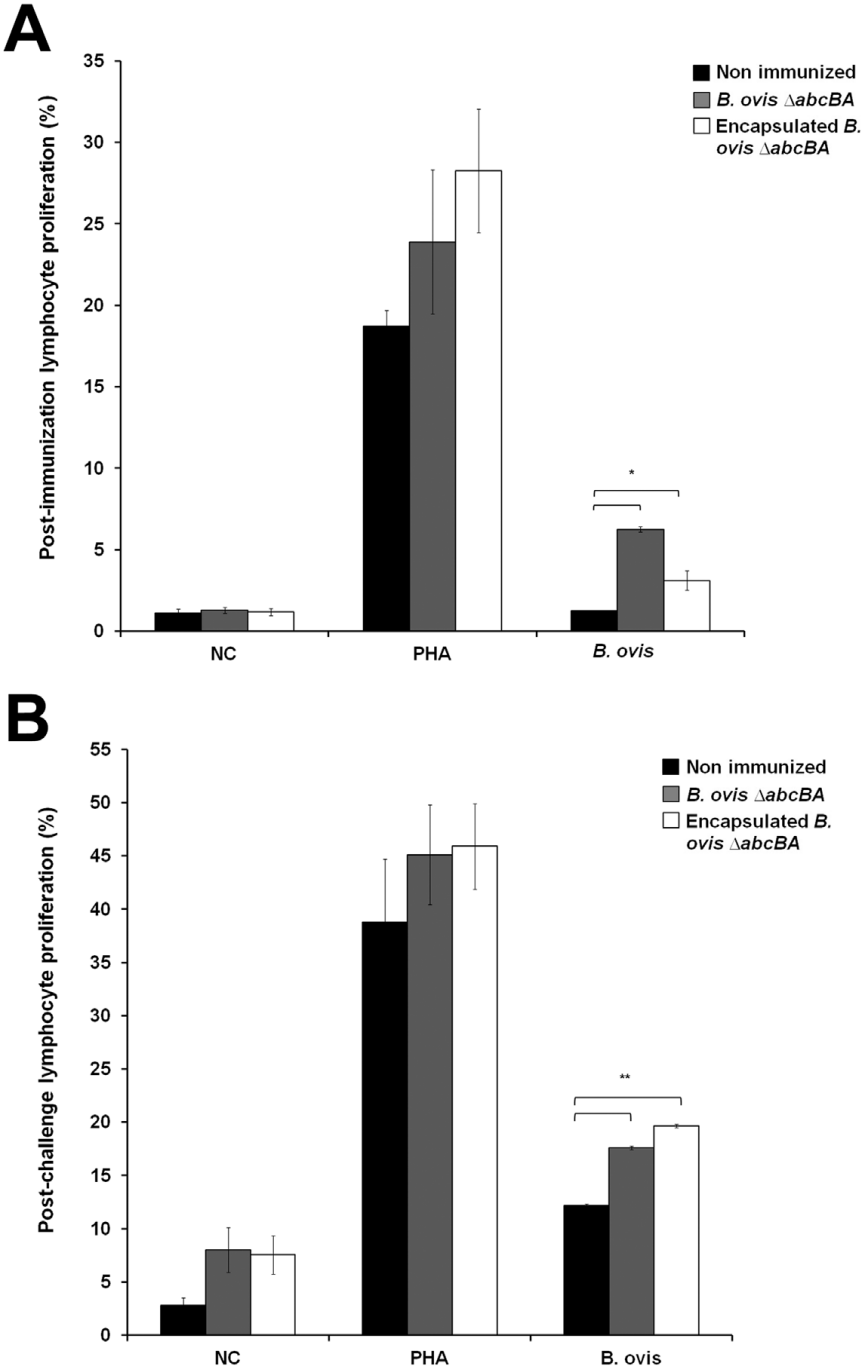
Lymphocyte proliferation assay at 8 weeks post immunization (A) and 8 weeks post challenge (B) in non immunized rams, and rams immunized with encapsulated or non encapsulated *Brucella ovis* Δ*abcBA*. Columns represent the mean of 10 rams. Data represent mean and standard error. Asterisks indicate statistical differences between groups (* p<0.05; ** p<0.01).

A proliferation assay was performed to evaluate the cellular immune responses. There was a significant increase in the percentage of lymphocyte proliferation in rams immunized with encapsulated or non-encapsulated *B*. *ovis* Δ*abcBA* before challenge (p<0.05) (Fig 3A) and after challenge (p<0.05 and p<0.01, respectively) (Fig 3B).

**Fig 4:**
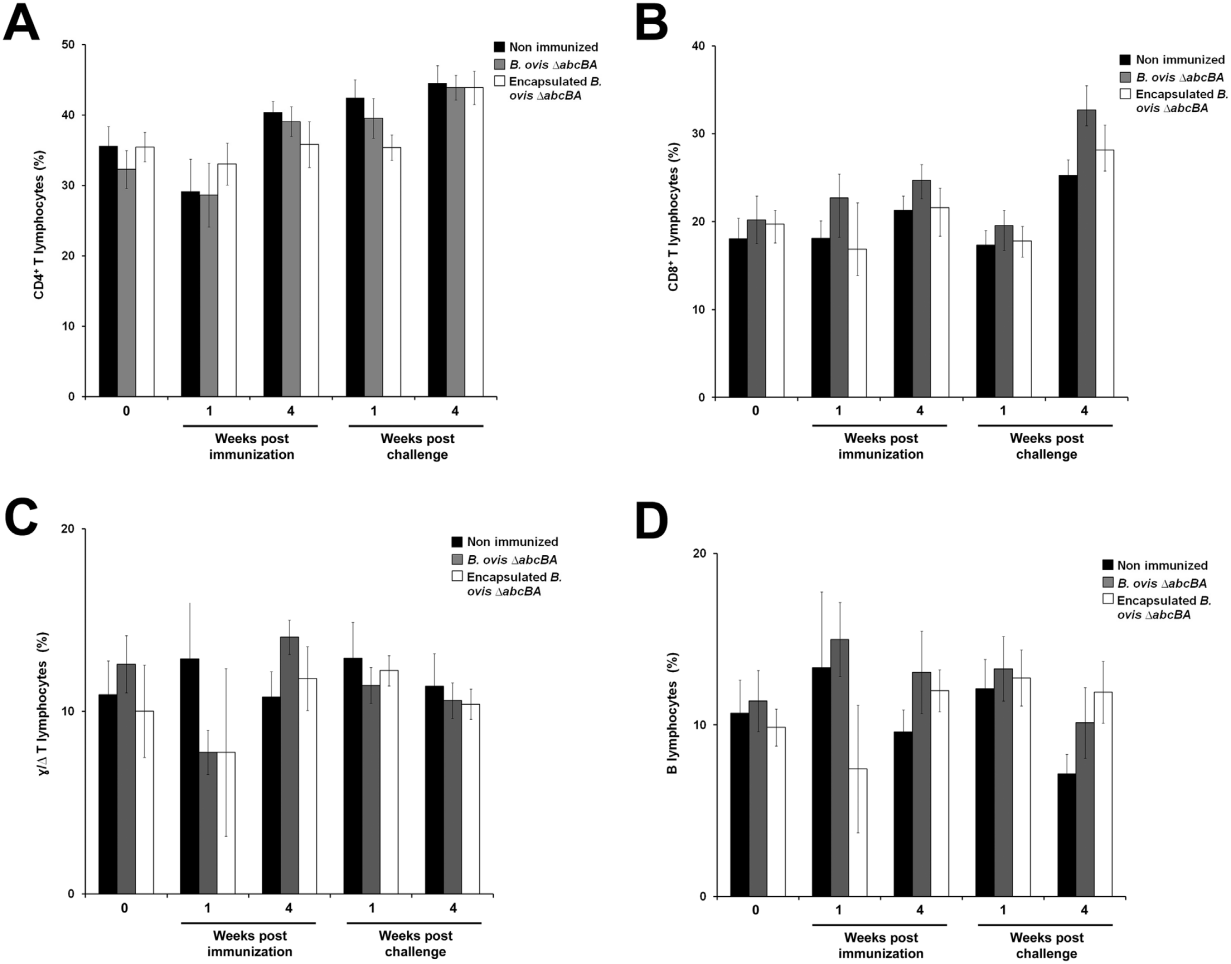
Peripheral blood leukocyte immunophenotyping of non immunized rams, and rams immunized with encapsulated or non encapsulated *B*. *ovis* Δ*abcBA*. Samples were obtained prior to immunization, 1 and 4 weeks after immunization, and 1 and 4 weeks after challenge. (A) CD4^+^ T lymphocytes, (B) CD8^+^ T lymphocytes, (C) γ/Δ T lymphocytes, and (D) B lymphocytes. The number of weeks before and after challenge is indicated in the x axis. Data represents mean and standard error.

Immunophenotyping of peripheral blood leukocytes indicated that there were no statistically significant differences among the groups, neither before nor after challenge (Fig 4).

### Immunization with *Brucella ovis* ΔabcBA prevented shedding of wild type *B*. *ovis* in experimentally challenged rams

**Fig 5:**
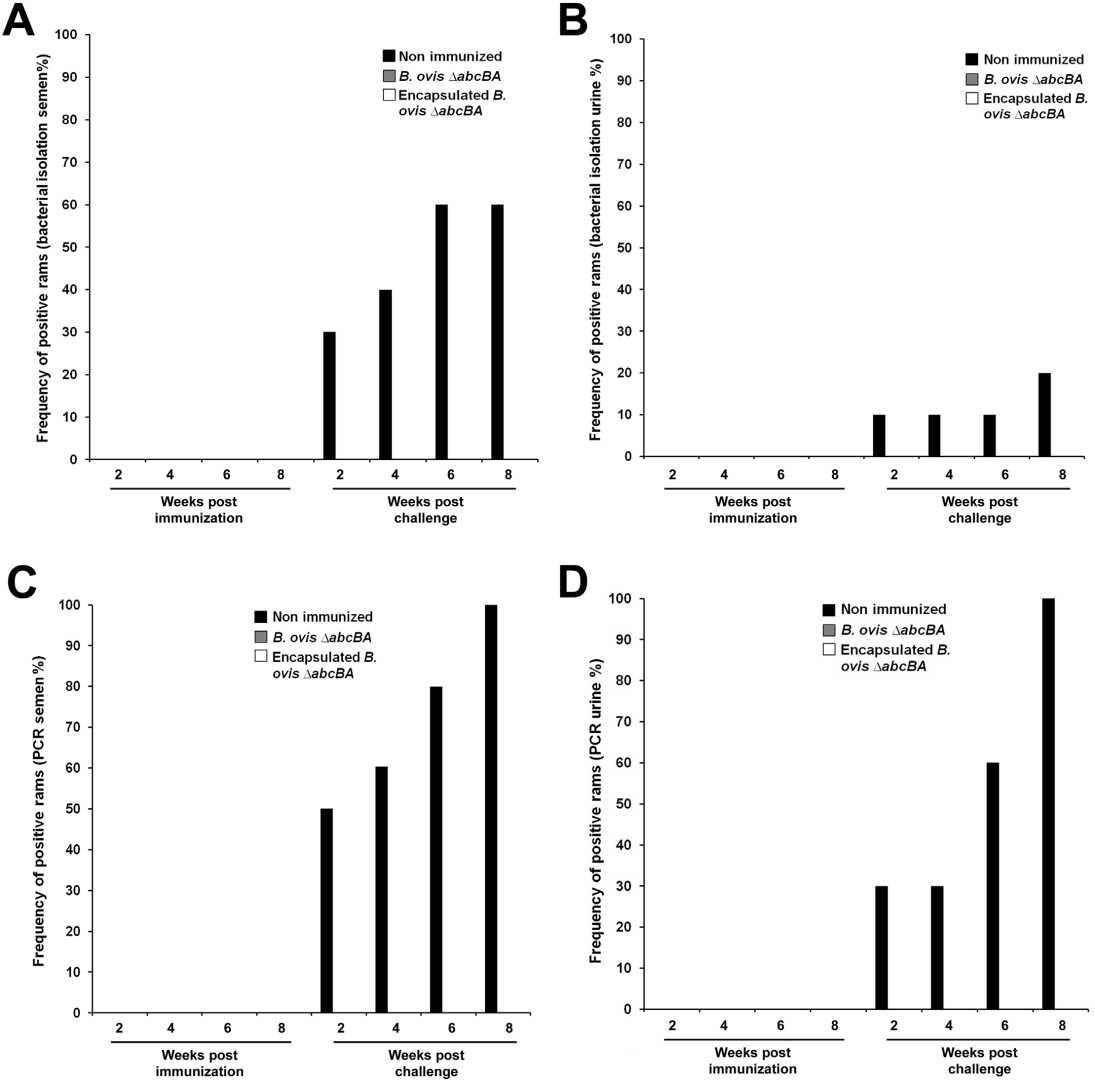
Frequency of isolation of wild type *Brucella ovis* from semen (A) and urine (B); or PCR detection in semen (C) or urine (D) samples, before and after challenge. The number of weeks is indicated in the x axis.

To assess protection induced by the *B*. *ovis* Δ*abcBA* strain, semen and urine samples were collected and processed for bacterial culture and PCR. During the eight weeks after immunization, there was no bacterial growth from semen or urine samples from rams immunized with encapsulated or non encapsulated *B*. *ovis* Δ*abcBA*. After challenge, none of the rams immunized with encapsulated or non encapsulated *B*. *ovis* Δ*abcBA* shed wild type *B*. *ovis* or *B*. *ovis* Δ*abcBA* in the semen or urine. Only non-immunized rams shed wild type *B*. *ovis* in the semen and urine after challenge (Fig 5A and 5B). Furthermore, there was no detectable *B*. *ovis* DNA in semen or urine samples from rams immunized with encapsulated or non encapsulated *B*. *ovis* Δ*abcBA*. *B*. *ovis* DNA was detected only in semen and urine samples from non immunized rams (Fig 5C and 5D).

### Immunization with *Brucella ovis* ΔabcBA prevented tissue colonization by wild type *B*. *ovis*

**Fig 6:**
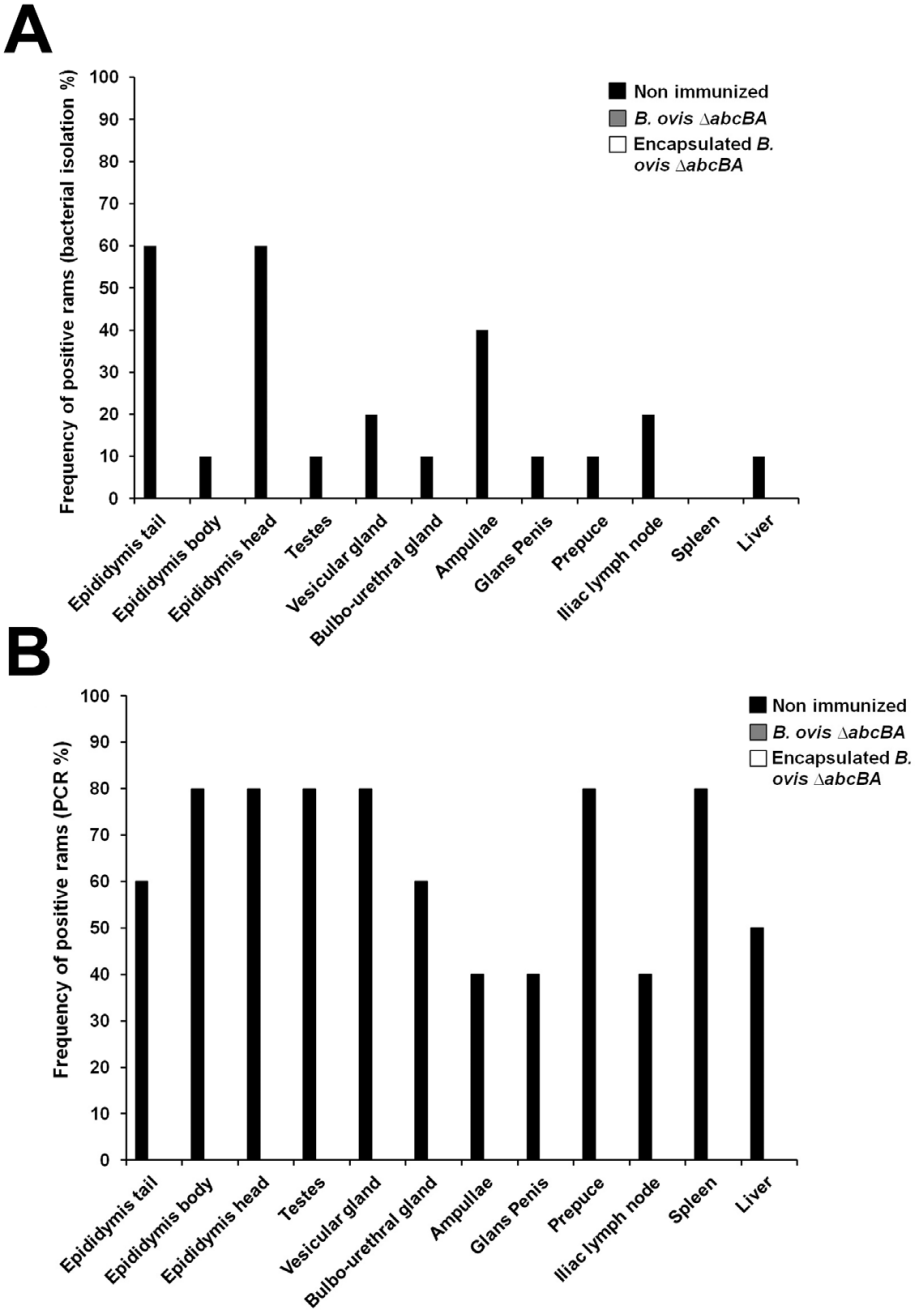
Frequency of isolation (A) or PCR detection (B) of wild type *Brucella ovis* in tissues from non immunized rams or rams immunized with encapsulated or non encapsulated *Brucella ovis* Δ*abcBA* at eight weeks post experimental challenge with wild type *B*. *ovis*.

Bacterial culture and PCR were performed with tissues of reproductive system, liver, spleen, and iliac lymph node. Wild type *B*. *ovis* or *B*. *ovis* Δ*abcBA* were not detected by bacterial culture from any of tissue samples collected from rams immunized with encapsulated or non encapsulated *B*. *ovis* Δ*abcBA*. In contrast, *B*. *ovis* was isolated with variable frequencies from all tissues from non-immunized rams, with the exception of the spleen (Fig 6A). Furthermore, there was no detection of *B*. *ovis* or *B*. *ovis* Δ*abcBA* DNA by PCR in tissue samples from rams immunized with encapsulated or non encapsulated *B*. *ovis* Δ*abcBA*. In contrast, 80% (8/10) of non-immunized rams had positive samples of the head and body of the epididymis, testes, vesicular gland, prepuce, and spleen (Fig 6B).

### Immunization with encapsulated *Brucella ovis* ΔabcBA prevented shedding of neutrophils in the semen in experimentally challenged rams

To evaluate the presence of inflammatory cells in the semen, smears were prepared with semen samples obtained after immunization and after challenge. Inflammatory cells were not observed in any of the semen samples from all rams prior to challenge, even after immunization with encapsulated or non encapsulated *B*. *ovis* **Δ** *abcBA*. At two weeks after challenge, most of the non-immunized rams (8/10) shed variable amounts of inflammatory cells in the ejaculate (predominantly neutrophils). Five rams (5/10) immunized with non-encapsulated *B*. *ovis* **Δ** *abcBA* shed inflammatory cells in the semen, but in most of these cases, with lower numbers when compared to non immunized rams. Importantly, only one ram vaccinated with encapsulated *B*. *ovis* **Δ** *abcBA* at one single time point shed lymphocytes and plasma cells in the semen, which differs from the *B*. *ovis*-induced leukospermia, which is characterized mostly by neutrophils (Table 1). Therefore, vaccination with encapsulated *B*. *ovis* **Δ** *abcBA* prevented shedding of neutrophils in the semen after infection with wild type *B*. *ovis*.

**Table 1:** Inflammatory cells in the semen of non immunized rams and rams immunized with encapsulated or non encapsulated *B*. *ovis* Δ*abcBA*, and challenged with wild type *B*. *ovis*. Semi-quantitative semen evaluation of inflammatory cells: (–) absence, (+) mild, (++) moderate, (+++) intense.

		Weeks post immunization				Weeks post challenge			
Group	Rams	2	4	6	8	2	4	6	8
Non Immunized	1	**-**	**-**	**-**	**-**	**+++**	**+++**	**+**	**++**
	2	**-**	**-**	**-**	**-**	**-**	**+**	**++**	**++**
	3	**-**	**-**	**-**	**-**	**+++**	**++**	**+**	**++**
	4	**-**	**-**	**-**	**-**	**+++**	**+++**	**+++**	**+++**
	5	**-**	**-**	**-**	**-**	**+**	**+**	**-**	**-**
	6	**-**	**-**	**-**	**-**	**+**	**+**	**+**	**+**
	7	**-**	**-**	**-**	**-**	**+**	**+**	**+**	**+**
	8	**-**	**-**	**-**	**-**	**+**	**++**	**-**	**-**
	9	**-**	**-**	**-**	**-**	**+**	**+++**	**+**	**++**
	10	**-**	**-**	**-**	**-**	**-**	**-**	**-**	**+++**
*B*. *ovis* Δ*abcBA*	11	**-**	**-**	**-**	**-**	**++**	**++**	**++**	**-**
	12	**-**	**-**	**-**	**-**	**-**	**-**	**-**	**-**
	13	**-**	**-**	**-**	**-**	**-**	**+++**	**+**	**++**
	14	**-**	**-**	**-**	**-**	**-**	**-**	**-**	**-**
	15	**-**	**-**	**-**	**-**	**-**	**-**	**-**	**-**
	16	**-**	**-**	**-**	**-**	**-**	**-**	**-**	**-**
	17	**-**	**-**	**-**	**-**	**-**	**-**	**-**	**-**
	18	**-**	**-**	**-**	**-**	+	++	+	+
	19	**-**	**-**	**-**	**-**	++	-	++	+
	20	**-**	**-**	**-**	**-**	+	+	+	+++
Encapsulated *B*. *ovis* Δ*abcBA*	21	**-**	**-**	**-**	**-**	**-**	**-**	**-**	**-**
	22	**-**	**-**	**-**	**-**	**-**	**-**	**-**	**-**
	23	**-**	**-**	**-**	**-**	**-**	**-**	**-**	**-**
	24	**-**	**-**	**-**	**-**	**-**	**-**	**-**	**-**
	25	**-**	**-**	**-**	**-**	**-**	**-**	**-**	**-**
	26	**-**	**-**	**-**	**-**	**-**	**-**	**-**	**-**
	27	**-**	**-**	**-**	**-**	**-**	**-**	**-**	**-**
	28	**-**	**-**	**-**	**-**	**-**	**-**	**-**	**-**
	29	**-**	**-**	**-**	**-**	**-**	**-**	**-**	**-**
	30	**-**	**-**	**-**	**-**	**-**	++	**-**	**-**

### Immunization with *Brucella ovis* ΔabcBA prevented the development of *B*. *ovis*-induced lesions in rams

**Fig 7:**
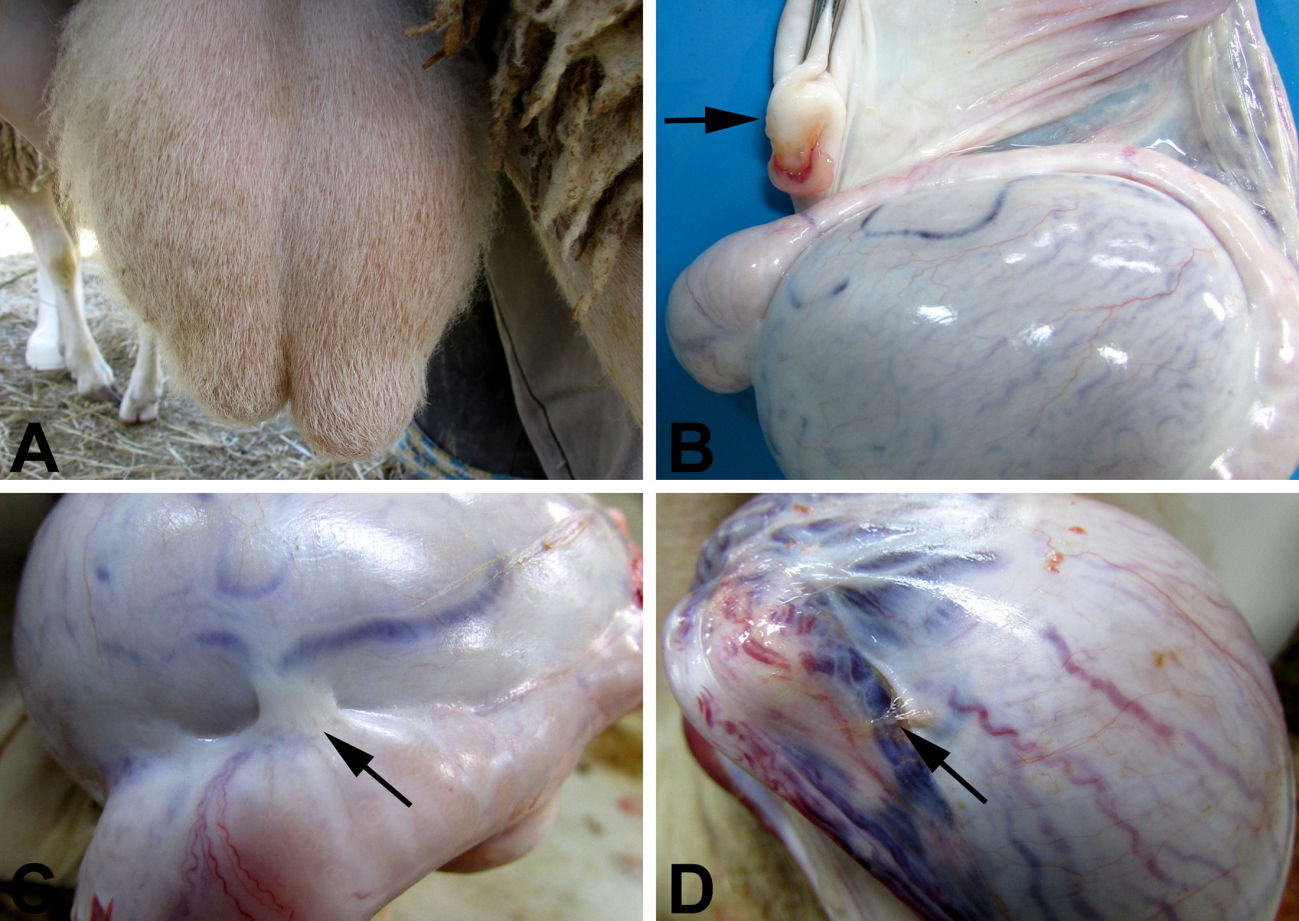
Gross lesions in the reproductive system of non immunized rams experimentally challenged with wild type *Brucella ovis*, at 8 weeks post infection. Asymmetry of the tail of the epididymis (A). Granuloma between the visceral and parietal layers of the tunica vaginalis, adjacent to the tail of the epididymis (black arrow) (B). Fibrous adhesion between testis and the head of the epididymis (black arrow) (C). Fibrinous adhesion on the tunica vaginalis (black arrow) (D).

At two weeks post-challenge, clinical examination indicated that three non-immunized rams developed unilateral swelling of epididymal tail (one ram developed this lesion in the right epididymis and the other two in the left), which was associated with pain on palpation, and caused asymmetry (Fig 7A). At two weeks post-challenge, it was possible to detect reduced testicular consistency during palpation, which tended to be flaccid (probably due to testicular degeneration), and nodular firm structures with approximately 2 x 2 cm, in the left epididymal tail of three non-immunized rams (interpreted as granulomas). Importantly, rams immunized with encapsulated or non encapsulated *B*. *ovis* **Δ** *abcBA* did not develop any clinical changes throughout the course of the experiment.

At necropsy, there were no lesions in rams immunized with encapsulated or non encapsulated *B*. *ovis* **Δ** *abcBA*. However, in three non-immunized rams (30%), there were round yellow-reddish and firm nodules with approximately 3 x 2 x 1 cm in the visceral surface of the tunica vaginalis, near the epididymis tail (Fig 7B). These rams had also fibrous (Fig 7C) and fibrinous (Fig 7D) adhesions between the tunica albuginea and segments of the epididymis, and edema in the tunica vaginalis.

**Fig 8:**
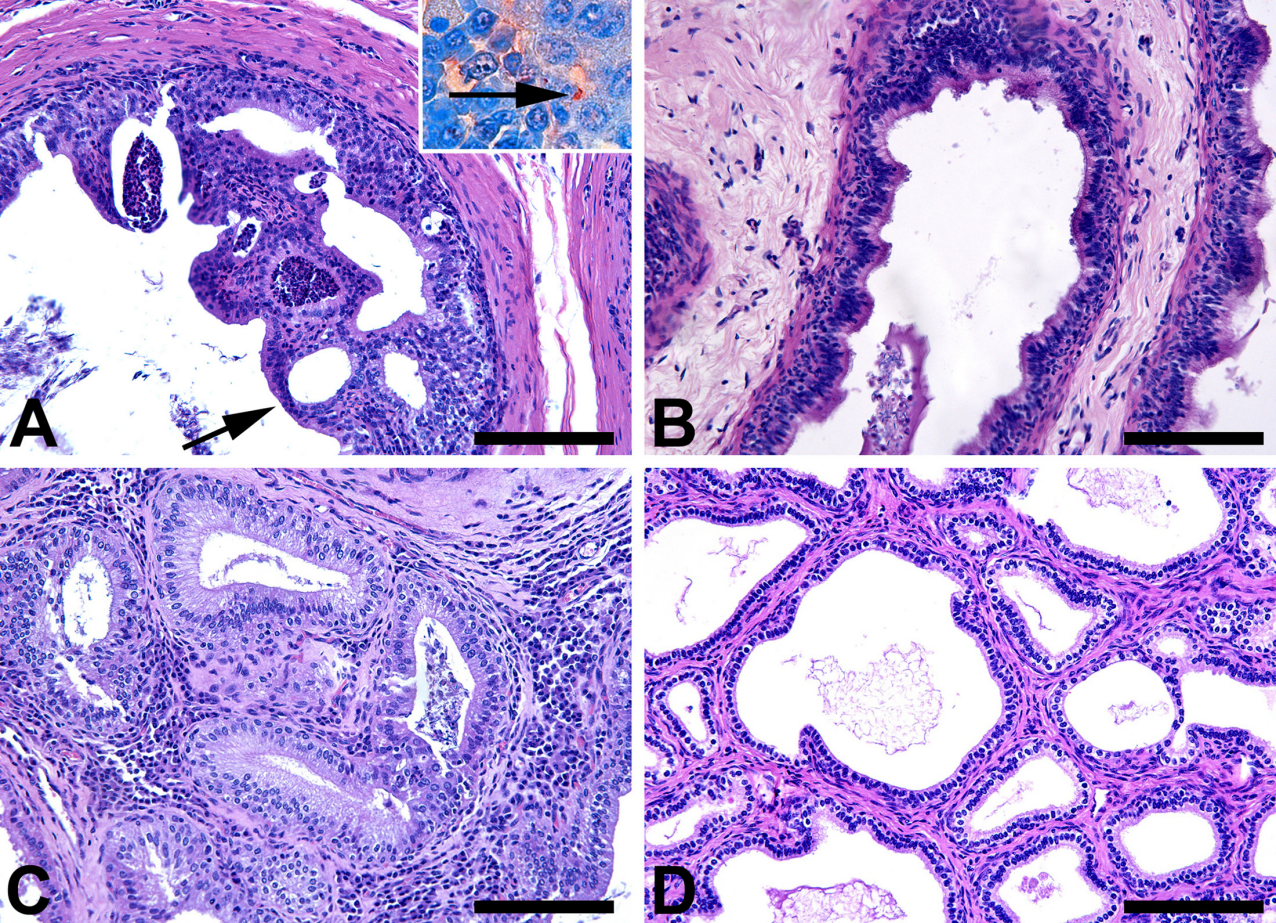
Microscopic changes in the reproductive system of non immunized rams experimentally challenged with *Brucella ovis*, at 8 weeks post challenge. Severe neutrophilic epididymitis associated to cystic epithelium degeneration (black arrow), with positive immunestaining for *B*. *ovis* (inset, 100X) in the tail of the epididymis from a non immunized ram (A). Tail of the epididymis from a ram immunized with encapsulated *B*. *ovis* Δ*abcBA* and challenged with wild type *B*. *ovis* with no histological changes (B). Moderate lympho-histiocytic and neutrophilic inflammatory infiltrate in the ampullae of a non immunized ram (C). Absence of histological changes in the ampullae of a ram immunized with encapsulated *B*. *ovis* Δ*abcBA* and challenged with wild type *B*. *ovis* (D), H. E. Bar = 50 μm.

Microscopically, there were moderate or intense inflammatory infiltrates composed of neutrophils, lymphocytes, and histiocytes, mainly in the tail (20%) (Fig 8), and head of the epididymis (20%), ampullae (20%) (Fig 8), vesicular gland (30%), iliac lymph nodes (70%), liver (10%), spleen (60%), and tunica vaginalis (20%) of non-immunized rams, with positive immunestaining for *B*. *ovis*. Minimal histopathological changes were observed in tissues from rams immunized with non-encapsulated *B*. *ovis* **Δ** *abcBA*, characterized by a mild lymphocytic inflammation in vesicular and bulbourethral glands. Importantly, there were no significant histological changes in rams immunized with encapsulated *B*. *ovis* **Δ** *abcBA* (Fig 8).

## Discussion

The *B*. *ovis* Δ*abcBA* strain has been originally characterized in the mouse model in which it is strongly attenuated and does not cause lethality in IRF-1 mice, which are killed when infected with wild type *B*. *ovis* [23]. Further studies demonstrated that inactivation of a putative species-specific ABC transporter interferes with expression of the *virB*-encoded type IV secretion system (T4SS) in a post transcriptional level [26]. Importantly, this T4SS is required for intracellular survival and *in vivo* persistence of *B*. *ovis* [29], and a *B*. *ovis virB* mutant strain [29] has a phenotype that is completely similar to the *B*. *ovis* Δ*abcBA* strain [23]. Interestingly, the *B*. *ovis* Δ*abcBA* strain induces wild type levels of humoral and cellular responses in rams, which are its preferential host [24]. These results prompted us to evaluate the *B*. *ovis* Δ*abcBA* strain as a vaccine candidate in the mouse, in which the alginate encapsulated vaccine induced significant protection against experimental challenge with wild type *B*. *ovis* [25]. This study demonstrated some exceptionally favorable results supporting the usefulness of the *B*. *ovis* Δ*abcBA* strain as a vaccine strain for protecting against *B*. *ovis* infection, transmission, and disease. This study confirmed that the *B*. *ovis* Δ*abcBA* strain is not shed in the semen or urine of vaccinated rams, and that it induces humoral and cellular immune responses. Most importantly, immunization with the *B*. *ovis* Δ*abcBA* strain prevented shedding of the wild type strain in the semen and urine after experimental challenge. Furthermore, this vaccination protocol, particularly with the vaccine strain encapsulated in alginate microparticules, resulted in prevention of: (i) infection (i.e. colonization of tissues by the wild type strain after challenge), (ii) secretion of the wild type strain in the semen and urine (possibly preventing transmission of the disease), and (iii) *B*. *ovis*-induced clinical and pathological changes in the genital tract. Importantly, to the best of our knowledge, this is the first report of development of a live attenuated and encapsulated *B*. *ovis* vaccine that is protective for rams.

In order to be considered a safe and effective *Brucella* spp. vaccine candidate, the vaccinal strain should not be pathogenic for the species to be immunized or to humans, it should not be shed in environment, and it should not interfere with serological tests [30]. Although *B*. *melitensis* Rev-1 vaccine strain induces some level of cross protection against *B*. *ovis*, it has residual pathogenic potential for animals, it is capable of infecting and causing human disease, and it interferes with routinely used serological tests for diagnosing *B*. *melitensis* infection [30]. Therefore, the *B*. *ovis* Δ*abcBA* strain may be a safe and effective vaccine against *B*. *ovis* infection in rams, since it does not have any pathogenic potential for rams, it does not cause disease in humans, and it does not interfere with routine diagnostic tests for diagnosis of *B*. *melitensis* infection since *B*. *ovis* has a rough lypopolysaccharide (LPS) [31].

Original results clearly demonstrated that the *B*. *ovis* Δ*abcBA* strain was strongly attenuated in the mouse [23] and in rams [24], which could potentially impair its potential as a vaccine candidate due to the lack of persistence in the host. Indeed, in the mouse model, the *B*. *ovis* Δ*abcBA* strain encapsulated in alginate microcapsules induced better protection than the same non encapsulated strain [25]. Live vaccines associated with a slow release vehicle tend to be more efficient, and therefore these vehicles are considered a new generation of adjuvants [32]. Alginate is a natural and biologically compatible biopolymer that has been used to develop vaccine vehicles [33]. Numerous studies have demonstrated that the use of this delivery system is quite efficient for proteins such as insulin, chemokines, and erythropoietin [34–36]. Synthetic polymers (e.g. poly-caprolactone and poly-lactide-co-glycolide) have also been used for vaccine encapsulation [37,38], but results obtained with *B*. *melitensis* and *B*. *abortus* encapsulation with alginate have been very promising, showing increased protection and immunogenicity [39–41]. Encapsulation of the *B*. *ovis* Δ*abcBA* in this study, aiming a slower released of the vaccine strain in the subcutaneous site of injection, induced a better performance of the vaccine strain. Interestingly, our unpublished preliminary results demonstrated that encapsulated *B*. *ovis* Δ*abcBA* indeed persists longer in the mouse and it is associated with an evident inflammatory reaction at the subcutaneous site of injection, which is absent in the site of injection of non encapsulated *B*. *ovis* Δ*abcBA* [25].

Under field conditions, a simple, inexpensive, and widely used approach to screen for *B*. *ovis* infection is through semen evaluation. *B*. *ovis* infection induces secretion of inflammatory cells, mainly neutrophils, in the semen, although other bacteria such as *Actinobacillus seminis* and *Histophilus somni* may also cause similar changes [5,42]. All non-immunized rams shed neutrophils in the semen after challenge, whereas five out of ten rams immunized with non-encapsulated *B*. *ovis* Δ*abcBA* shed small numbers of neutrophils in the semen. Importantly, none of the rams immunized with the encapsulated *B*. *ovis* Δ*abcBA* shed neutrophils in the semen. At one single time point after challenge, one ram immunized with the encapsulated *B*. *ovis* Δ*abcBA* shed lymphocytes and plasma cells in moderate amounts in the semen, which is likely to be an occasional finding, not caused *B*. *ovis* infection since experimental infections with *B*. *ovis* are associated with secretion of neutrophils in the semen [5]. These results indicate that encapsulation improved the protection induced by *B*. *ovis* Δ*abcBA* strain. Importantly, should this vaccine strain be used under field conditions, these data support the notion that vaccination will not interfere with screening of infected rams by semen evaluation.

Experimentally or naturally infected rams often eliminate *B*. *ovis* in the semen and urine, which is thought to be the most important form of transmission of the disease [24,27,43]. The immunization protocol with encapsulated *B*. *ovis* Δ*abcBA* developed in this study prevented shedding of wild type *B*. *ovis* in the semen and urine, as demonstrated by bacterial culture and a previously described species-specific PCR protocol [27]. Therefore, our data support the notion that this vaccination protocol is a useful tool to mitigate risk of *B*. *ovis* transmission within the flock.

According to Carvalho Junior *et al* [5], gross lesions are evident in epididymal tail and vesicular gland of rams experimentally infected with *B*. *ovis*. Naturally infected rams develop similar changes, and the most frequently affected organs are the epididymis and vesicular gland [44], and these results are consistent with our findings. Vaccination with *B*. *ovis* Δ*abcBA* prevented development of clinical changes as well as gross or microscopic lesions in experimentally challenged rams.

Although encapsulated *B*. *ovis* Δ*abcBA* had a better performance as a vaccine candidate when compared to the non encapsulated vaccine, lymphocyte proliferation did not differ between these two vaccine formulations. Interestingly, Arenas-Gamboa *et al* [39], evaluating cellular immune response in deer, demonstrated that *B*. *abortus* RB51 encapsulated with alginate induces significantly higher cellular immune response when compared to non-encapsulated RB51. No significant changes in peripheral blood leukocyte profiles were observed in this study, which contrasts with our previous findings [24]. It is possible that lack of reproducibility of these results may be related to variation in the genetic background of rams used in different studies since a higher variation in immune resposes are expected in outbread animals when compared to inbreed mice.

## Conclusion

*B*. *ovis* Δ*abcBA* encapsulated with sterile alginate is immunogenic and confers protection against *B*. *ovis* experimental infection in rams. This vaccination protocol prevented infection, secretion of wild type *B*. *ovis* in the semen and urine, shedding of neutrophils in the semen, and the development of clinical changes, gross and microscopic lesions induced by the wild type *B*. *ovis* reference strain. Collectively, our data indicated that the *B*. *ovis* Δ*abcBA* strain is an exceptionally good vaccine strain for preventing brucellosis caused by *B*. *ovis* infection in rams.

## References

[1] Franco MP, Mulder M, Gilman RH. Human brucellosis. Lancet Infect Dis. 2007;7: 775–786. 18045560 10.1016/S1473-3099(07)70286-4

[2] Santos RL, Martins TM, Borges AM, Paixão TA. Economic losses due to bovine brucellosis in Brazil. Pesq Vet Bras. 2013;33: 759–764.

[3] Garrity GM. Bergey’s Manual of Systematic Bacteriology. 2nd ed Springer Press, New York; 2001.

[4] Poester FP, Samartino LE, Santos RL. Pathogenesis and pathobiology of brucellosis in livestock. Rev Sci Tech. 2013;32: 105–115. 23837369 10.20506/rst.32.1.2193

[5] Carvalho CA Junior, Moustacas VS, Xavier MN, Costa EA, Costa LF, Silva TMA, et al Andrological, pathologic, morphometric, and ultrasonographic findings in rams experimentally infected with *Brucella ovis* . Small Rumin Res. 2012;102: 213–222.

[6] Blasco JM. *Brucella ovis* In: Nielsen K, Duncan JR(Eds), *Animal Brucellosis*. CRC Press, Boca Raton, FL; 1990, p. 351–378.

[7] Thoen CO, Enright F, Cheville NF. *Brucella* IN: Gyles CL, Thoen CO, eds. Pathogenesis of bacterial infections in animals. Ames: Iowa State University Press; 1993 p. 236–247.

[8] Sancho P, Tejedor C, Sidhu-Muñoz R, Lago-Fernández L, Vizcaíno N. Evaluation in mice of *Brucella ovis* attenuated mutants for use as live vaccines against *B*. *ovis* . Vet Res. 2014;45: 61–71. 10.1186/1297-9716-45-61 24898325 PMC4057616

[9] Soler-Lloréns P, Gil-Ramírez Y, Zabalza-Baranguá A, Iriarte M, Conde-Álvarez R, Zúñiga-Ripa A, et al Mutants in the lipopolysaccharide of *Brucella ovis* are attenuated and protect against *B*. *ovis* infection in mice. Vet Res. 2014;45: 2–11. 25029920 10.1186/s13567-014-0072-0 PMC4107470

[10] Estein SM, Fiorentino MA, Paolicchi FA, Clausse M, Manazza J, Cassataro J, et al The polymeric antigen BLSOmp31 confers protection against *Brucella ovis* infection in rams. Vaccine. 2009;27: 6704–6711. 10.1016/j.vaccine.2009.08.097 19748579

[11] Cassataro J, Pasquevich KA, Estein SM, Laplagne DA, Velikovsky CA, De la Barrera S, et al A recombinat subunit vaccine based on the insertion of 27 aminoacids from Omp31 to the N-terminous of BLS induced a similar degree of protection against *B*. *ovis* than Rev 1 vaccination. Vaccine. 2007;25: 4437–4446. 17442465 10.1016/j.vaccine.2007.03.028

[12] Corbel MJ (ed). Brucellosis in humans and animals, Geneva, World Health Organization, 2006.

[13] Avila-Calderón ED, Lopez-Merino A, Sriranganathan N, Boyle SM, Contreras-Rodríguez A. A History of the development of *Brucella* vaccines. BioMed Res Int. 2013;2013: 743509 10.1155/2013/743509 23862154 PMC3686056

[14] Olsen SC. Recent developments in livestock and wildlife brucellosis vaccination. Rev Sci Tech. 2013;32: 207–217. 23837378 10.20506/rst.32.1.2201

[15] Marín CM, Barberán M, Jiménez De Bagués MP, Blasco JM. Comparison of subcutaneous and conjunctival routes of Rev 1 vaccination for the prophylaxis of *Brucella ovis* infection in rams. Res Vet Sci. 1990;48: 209–215. 2110377

[16] Blasco JM. A review of the use of *B*. *melitensis* Rev 1 vaccine in adult sheep and goats. Prev Vet Med. 1997;31: 275–283. 9234451 10.1016/s0167-5877(96)01110-5

[17] Grilló MJ, Bosseray N, Blasco JM. In vitro markers and biological activity in mice of seed lot strains and commercial *Brucella melitensis* Rev.1 and *Brucella abortus* B19 vaccines. Biologicals. 2000;28: 119–127. 10885618 10.1006/biol.2000.0249

[18] Lantier F, Fensterbank R. Kinetics of Rev.1 infection in sheep In: Plommet M, Verger JM. (Eds.), *Brucella melitensis*. Martinus Niijhoff, Dordrecht; 1985, 247–251.

[19] Robinson A. Guidelines for coordinated human and animal brucellosis surveillance In FAO ANIMAL PRODUCTION AND HEALTH PAPER 156, 2003.

[20] Vershilova PA. The use of live vaccine for vaccination of human beings against brucellosis in the USSR. Bull World Health Organ. 1961;24: 85–89. 13780996 PMC2555373

[21] Davis DS, Elzer PH. *Brucella* vaccines in wildlife. Vet Microbiol. 2002;90: 533–544. 12414169 10.1016/s0378-1135(02)00233-x

[22] Schurig GG, Sriranganathan N, Corbel MJ. Brucellosis vaccines: past, presentand future. Vet Microbiol. 2002;90: 479–496. 12414166 10.1016/s0378-1135(02)00255-9

[23] Silva TMA, Paixão TA, Costa EA, Xavier MN, Sá JC, Moustacas VS, et al Putative ATP-binding cassette transporter is essential for *Brucella ovis* pathogenesis in mice. Infect Immun. 2011;79: 1706–1717. 10.1128/IAI.01109-10 21300772 PMC3067543

[24] Silva APC, Macêdo AA, Costa LF, Turchetti AP, Bull V, Pessoa MS, et al *Brucella ovis* lacking a species-specific putative ATP-binding cassete transporter is attenuated but immunogenic in rams. Vet Microbiol. 2013;167: 546–553. 10.1016/j.vetmic.2013.09.003 24075357

[25] Silva APC, Macêdo AA, Silva TMA, Ximenes LCA, Brandão HM, Paixão TA, et al Protection of an encapsulated live attenuated strain of *Brucella ovis* against experimental challenge in the murine model. Clin Vaccine Immunol. 2015, in press. 10.1128/CVI.00191-15 PMC4478529 25947146

[26] Silva TMA, Mol JPS, Winter MG, Atruli V, Xavier MN, Pires SF, et al The predicted ABC transporter AbcEDCBA is required for type IV secretion system expression and lysosomal evasion by *Brucella ovis* . Plos One. 2014;12: e114532. 10.1371/journal.pone.0114532 PMC4256435 25474545

[27] Xavier MN, Silva TMA, Costa EA, Paixão TA, Moustacas VS, Carvalho CA Junior, et al Development and evaluation of a species-specific PCR assay for the detection of *Brucella ovis* infection in rams. Vet Microbiol. 2010;145: 158–164. 10.1016/j.vetmic.2010.02.037 20347534

[28] Matrone M, Keid LB, Rocha VCM, Vejarano MP, Ikuta CY, Rodriguez CA, et al Evaluation of DNA extraction protocols for *Brucella abortus* pcr detection in aborted fetuses or calves born from cows experimentally infected with strain 2308. Braz J Microbiol. 2009;40: 480–489. 24031391 10.1590/S1517-838220090003000010 PMC3768545

[29] Sá JC, Silva TMA, Costa EA, Silva APC, Tsolis RM, Paixão TA, et al The *virB* encode type IV secretion system is critical for establishment of infection and persistence of *Brucella ovis* infection in mice. Vet Microbiol. 2012;159: 130–140. 10.1016/j.vetmic.2012.03.029 22483850

[30] Banai M. Control of small ruminant brucellosis by use of *Brucella melitensis* Rev. 1 vaccine: laboratory aspects and field observations. Vet Microbiol. 2002;90: 497–519. 12414167 10.1016/s0378-1135(02)00231-6

[31] Nielsen K. Diagnosis of brucellosis by serology. Vet Microbiol. 2002;90: 447–459. 12414164 10.1016/s0378-1135(02)00229-8

[32] Hanes J, Cleland JL, Langer R. New advances in microsphere based single-dose vaccines. Adv Drug Del Rev. 1997;28: 97–117. 10.1016/s0169-409x(97)00053-7 10837567

[33] Blandino A, Macías M, Cantero D. Formation of calcium alginate gel capsules: influence of sodium alginate and CaCl_2_ concentration on gelation kinetics. J Biosci Bioeng. 1999;88: 686–689. 16232687 10.1016/s1389-1723(00)87103-0

[34] Marschutz MK, Bernkop-Schnurch A. Oral peptide drug delivery: polymer-inhibitor conjugates protecting insulin from enzymatic degradation *in vitro* . Biomaterials. 2000;21: 1499–1507. 10872779 10.1016/s0142-9612(00)00039-9

[35] Takenaga M, Yamaguchi Y, Kitagawa A, Ogawa Y, Mizushima Y, Igarashi R. A novel sustained-release formulation of insulin with dramatic reduction in initial rapid release. J Control Release. 2002;79: 81–91. 11853920 10.1016/s0168-3659(01)00518-1

[36] Qiu B, Stefanos S, Ma J, Lalloo A, Perry BA, Leibowitz MJ, et al A hydrogel prepared by in situ cross-linking of a thiol-containing poly(ethylene glycol)-based copolymer: a new biomaterial for protein drug delivery. Biomaterials. 2003;24: 11–18. 12417173 10.1016/s0142-9612(02)00227-2

[37] Visscher GE, Robinson RL, Argentieri GI. Tissue response to biodegradable injectable microcapsules. J Biomater Appl. 1987;2: 118–131. 3333063 10.1177/088532828700200103

[38] Murillo M, Grilló MJ, Reñé J, Marín CM, Barberán M, Goñi MM, et al A *Brucella ovis* antigenic complex bearing poly-epsilon-caprolactone microparticles confer protection against experimental brucellosis in mice. Vaccine. 2001;19: 4099–4106. 11457533 10.1016/s0264-410x(01)00177-3

[39] Arenas-Gamboa AM, Ficht TA, Kahl-Mcdonagh MM, Rice-Ficht AC. Immunization with a single dose of a microencapsulated *Brucella melitensis* mutant enhances protection against wild-type challenge. Infect Immun. 2008;76: 2448–2455. 10.1128/IAI.00767-07 18362129 PMC2423109

[40] Arenas-Gamboa AM, Ficht TA, Davis DS, Elzer PH, Gonzalez AW, Rice-Ficht AC. Enhanced immune response of red deer (*Cervus elaphus*) to live RB51 vaccine strain using composite microspheres. J Wild Dis. 2009;45: 165–173. 10.7589/0090-3558-45.1.165 PMC3350799 19204345

[41] Arenas-Gamboa AM, Ficht TA, Kahl-McDonagh MM, Gomez G, Rice-Ficht AC. The *Brucella abortus* S19 Δ*vjbR* live candidate is safer than S19 and confers protection against wild-type vaccine challenge in BALB/c mice when delivered in a sustained-release vehicle. Infect Immun. 2009;77: 877–884. 10.1128/IAI.01017-08 19047401 PMC2632017

[42] Moustacas VS, Silva TMA, Costa LF, Carvalho CA Junior, Santos RL, Paixão TA. Clincal and pathological changes in rams experimentally infected with *Actinobacillus seminis* and *Histophilus somni* . Sci World J. 2014;2014: 241452. 10.1155/2014/241452 PMC3925577 24592151

[43] Costa EA, Sant'Ana FM, Carvalho CA Junior, Moustacas VS, Silva SMMS, Paixão TA, et al Diagnosis of Brucella ovis infection by serology and PCR in urine samples from naturally infected rams in the state of Piauí. Arq Bras Med Vet Zootec. 2012;64: 751–754.

[44] Jansen BC. The pathology of bacterial infection of the genitalia in rams. J Vet Res. 1980;47: 263–267. 7231922

